# Characterization of Subtilin L-Q11, a Novel Class I Bacteriocin Synthesized by *Bacillus subtilis* L-Q11 Isolated From Orchard Soil

**DOI:** 10.3389/fmicb.2019.00484

**Published:** 2019-03-15

**Authors:** Yuxuan Qin, Yao Wang, Yinghao He, Ying Zhang, Qianxuan She, Yunrong Chai, Pinglan Li, Qingmao Shang

**Affiliations:** ^1^Beijing Advanced Innovation Center for Food Nutrition and Human Health, College of Food Science and Nutritional Engineering, Key Laboratory of Functional Dairy, China Agricultural University, Beijing, China; ^2^Department of Biology, Northeastern University, Boston, MA, United States; ^3^Key Laboratory of Biology and Genetic Improvement of Horticultural Crops, The Institute of Vegetables and Flowers, Chinese Academy of Agricultural Sciences, Beijing, China

**Keywords:** Subtilin L-Q11, *Bacillus subtilis*, bacteriocin, antibacterial activity, antibacterial mechanism

## Abstract

Bacteriocins are peptides or proteins synthesized by bacterial ribosomes that show killing or inhibitory activities against different groups of bacteria. Bacteriocins are considered potential alternatives to traditional antibiotics, preservatives in pharmaceutical and food industries. A strain L-Q11 isolated from orchard soil was phylogenetically characterized as *Bacillus subtilis* based on 16S rRNA gene sequencing analysis. A novel class I bacteriocin (Subtilin L-Q11), was identified and purified from L-Q11 cell-free supernatant in a four-step procedure, including salt precipitation, cation exchange, gel filtration, and reverse-phase high-performance liquid chromatography (RP-HPLC). The molecular mass (3,552.9 Da) of this novel bacteriocin was determined by Matrix-assisted laser desorption/ionization time-of-flight mass spectrometry (MALDI-TOF-MS). The purified Subtilin L-Q11 exhibited optimal features in pH tolerance, thermostability, and sensitivity to proteases. Further, Subtilin L-Q11 showed inhibitory activities against a number of bacteria including some human pathogens and food spoilage bacteria, in particular *Staphylococcus aureus.* All these important features make this novel bacteriocin a potential candidate for the development of a new antibacterial drug or food preservative in the future.

## Introduction

*Bacillus subtilis* is a Gram-positive, spore-forming soil bacterium, which is known to produce more than twenty different, structurally diverse antimicrobial compounds (Stein, 2005). The majority of these compounds are peptide-based antibiotics. There are two principal synthetic pathways for biosynthesis of peptide-based antibiotics in *B. subtilis*: (i) non-ribosomal synthesis of peptides by non-ribosomal peptide synthetases (NRPSs), and (ii) ribosomally synthesized antimicrobial peptides (Giessen and Marahiel, 2012).

Bacteriocins are ribosomally synthesized peptide antibiotics, which show antagonistic activities toward different bacteria, especially to those that are genetically close to the producing strains (Klaenhammer, 1988). Bacteriocins are often employed as a weapon by the producing bacteria to compete and protect themselves in the natural environment. In the food industry, bacteriocins produced by lactic acid bacteria have long been applied in food preservation (Tagg et al., 1976). Bacteriocins also demonstrate great potentials as antimicrobial compounds in pharmaceutical, agricultural, and biochemical engineering industries (Dischinger et al., 2014). Bacteriocins have become more popular in recent years due to their therapeutic effects in treating bacterial infection, even against certain multidrug resistant bacteria (Garneau et al., 2002; Cotter et al., 2005). Furthermore, compared to chemically synthesized traditional antibiotics or food preservatives, bacteriocins present low toxicity toward human hosts because human cells do not possess a receptor recognized by bacteriocins (Kanmani et al., 2013; Yang et al., 2014). Bacteriocins were originally only found to be synthesized by *Lactobacillus*. It is now believed that more than 99% of bacteria can produce at least one type of bacteriocins, however, the majority of them are yet to be identified (Riley and Wertz, 2002).

An average of 4 and 5% of the *B. subtilis* genome is believed to be involved in the biosynthesis of various antimicrobial compounds (Stein, 2005). Thus, species from the *Bacillus* genus represent a great pool for screen and discovery of novel bacteriocins (Phelan et al., 2013). Based on distinct structural and functional characteristics, bacteriocins can be divided into different classes. Class I bacteriocins are also named lantibiotics. Most of the bacteriocins produced by *Bacillus* species belong to the class I bacteriocins (Singh et al., 2012).

In this study, we purified and characterized Subtilin L-Q11, a novel class I bacteriocin from *B. subtilis* L-Q11, a strain isolated from the orchard soil in Beijing, China. We demonstrated that this new bacteriocin has great potential to be used as a biologically synthesized antibiotic or food preservative in agricultural field and food industry. We also investigated the mode of action of this novel bacteriocin against the bacterial pathogen *S. aureus* ATCC 29213.

## Materials and Methods

### Bacterial Strains and Growth Conditions

The spore-forming strain L-Q11 is a bacteriocin-producing bacterium isolated from the orchard soil in Beijing, China. Briefly, in order to isolate the spore-forming bacteria from natural environment. Soil samples from orchard was heated to kill non-spore-forming mesophiles, and then plated on rich media after dilution by sterilized water and incubated aerobically at 30°C. Thermophiles would not grow at this temperature, and anaerobic spore-formers (e.g., *Clostridium*) will not grow aerobically. Other mesophilic aerobic endospore-formers (e.g., *Heliospirillum*) are phototrophic, scarce, and require lots of light for growth.

*Bacillus subtilis* was grown in Luria-Bertani (LB) broth at 37°C under the shaking condition (200 rpm). *Staphylococcus aureus* 29213 was used as an indicator strain for antibacterial activities of the Subtilin L-Q11 and it was also cultured in LB broth at 37°C.

### Phylogenetic Characterization of the Strain L-Q11

The bacteriocin-producing *B. subtilis* strain was phylogenetically identified by 16S rRNA gene sequencing analysis. Briefly, the genomic DNA of L-Q11 was prepared by using a bacterial genomic extraction kit (TianGen, Beijing, China) according to the manufacturer’s instructions. A region in the 16s rRNA gene was amplified by PCR using two universal primers (27F: 5′-AGAGTTTGATCMTGGCTCAG-3′ and 1492R: 5′-TACGGYTACCTTGTTACGACTT-3′) (Lane, 1991). Thermal cycling consisted of initial denaturation at 95°C for 10 min, followed by 30 cycles of denaturation at 95°C for 30 s, annealing at 50°C for 1 min, and elongation at 72°C for 1 min, and finally, at 72°C for 10 min for completion. The PCR product was sent for sequencing after purification using GeneJET Gel Extraction Kit (QIAGEN, Germany). The sequencing result was blasted against the NCBI GenBank database^1^. If the similarity of the 16s rRNA gene sequence of this strain valued >97% when compared with a certain species, we considered that this strain belonged to this species.

### Crude Bacteriocin Preparation and Antibacterial Activity Assay

*Bacillus subtilis* L-Q11 was inoculated in 50 mL of LB broth and grown overnight, 1% of the overnight culture was re-inoculated into 1 L of LB broth and incubated at 37°C under the shaking condition at 200 rpm. Samples were collected every 4 h to record the medium pH and cell optical density (O.D._600_). Meanwhile, cell-free supernatant (CFS) from each time point was used to test the antibacterial activity against an indicator strain (*S. aureus* ATCC 29213). Briefly, we centrifuged the culture samples at 12,000 rpm for 15 min at 4°C to remove the cell pellets. The supernatant was filter-sterilized by passing through the Nalgene^TM^ Rapid-Flow^TM^ Sterile Disposable Filter (0.22 μm, Thermo Fisher Scientific, MA, United States) to obtain the CFS. The antibacterial activity of the crude bacteriocin in the CFS was determined by measuring the diameter of the inhibition zone with vernier caliper using *S. aureus* ATCC 29213 as an indicator strain. The antibacterial activity was presented as an arbitrary unit per milliliter of culture medium (AU/mL) and one AU was defined as the reciprocal of the highest 2-fold dilution exhibiting a clear zone of inhibition of the indicator strain (Deraz et al., 2005).

### Purification of Subtilin L-Q11

CFS was first mixed with 70% (w/v) ammonium sulfate at 4°C overnight under the continuous stirring and then centrifuged at 9,000 *g* for 20 min at 4°C. After that, the precipitation was collected and dissolved in 10 mL of PBS buffer (pH 7.0). The crude extract obtained after ammonium sulfate precipitation was applied onto the CM Sepharose Fast Flow cation-exchange column (16 mm × 200 mm, GE, Sweden) equilibrated and washed with 50 mM PBS (pH 6.0) linked to AKTA purifier 100 system (GE, Sweden). The elution was carried out by a linear gradient (from 0 to 1 M of NaCl) in the same buffer as equilibration for 60 min at a flow rate of 1 mL/min and monitored by an UV detector at the wavelength of 280 nm.

The active fractions obtained from the last step of purification were loaded onto a Sephadex G-10 (12 mm × 200 mm, GE, Sweden) column equilibrated with PBS buffer (pH 6.0) and connected to an AKTA purifier 100 system (GE, Sweden). The elution was carried out at the flow rate of 0.5 mL/min and recorded by UV at the wavelength of 280 nm by the same buffer.

The active fractions from above were applied onto a reverse phase high-performance liquid chromatography (RP-HPLC) system (Agilent, CA, United States) equipped with C18 reverse-phase column (5 μm, 4.6 mm × 250 mm, Agilent, United States) for further purification. The elution was performed using 5–95% linear gradient of acetonitrile containing 0.1% trifloroacetic acid (TFA) with a flow rate of 1 mL/min for 30 min and monitored by UV at the wavelength of 280 nm.

The collected fractions were concentrated and tested for antibacterial activities as described by An et al. (2017), and then stored at −80°C. The BCA kit (Thermo Fisher Scientific, MA, United States) was used for the protein concentration analysis.

### Molecular Weight and Amino Acid Sequence Determination

The molecular mass of the bacteriocin in the active fractions obtained after the HPLC purification were determined by matrix-assisted laser desorption ionization–time-of-flight (MALDI-TOF) mass spectrometry (MS) (Applied Biosystems, Foster city, CA, United States) in the positive mode. The active fraction was mixed together with the same volume of matrix solution contained 0.1% (v/v) of α-cyano-4-hydroxycinnamic acid (CHCA, Sigma, United States) dissolved in trifluoroacetic acid and 50% (v/v) of acetonitrile. For the MALDI analysis, 1 μl of the mixture was deposited directly onto the MALDI plate for drying. For identifying N-terminal amino sequence of Sublitin L-Q11, the purified Sublitin L-Q11 electrophoresed by SDS-PAGE was transferred to PVDF membrane (Millipore, United States). Residue amino sequence identified by Edman degradation was then compared with other published bacteriocin sequences by NCBI blast.

### Antimicrobial Spectrum Assay

To investigate the antimicrobial spectrum of Subtilin L-Q11, partially purified Subtilin L-Q11 preparation from cation exchange chromatography was adjusted to pH 6.0 by using 1 M NaOH, and the spectrum of antibacterial activity was determined against a series of food-borne and food-spoilage pathogens (Table 1) by using the pour plate method described by An et al. (2017).

**Table 1:** Antibacterial spectrum of Subtilin L-Q11.

Indictor strain	Source	Diameter of inhibition zone (mm)
**Gram-positive bacteria**		
*Bacillus amyloliquefaciens* ATCC 15841	ATCC	16.2 ± 0.2
*B. amyloliquefaciens* L-S60	Qin et al., 2015b	16.1 ± 0.3
*B. amyloliquefaciens* L-H15	Qin et al., 2015a	16.0 ± 0.3
*B. cereus* ATCC 14579	ATCC	16.4 ± 0.2
*Lactococcus lactis* NZ9000	Linares et al., 2010	9.4 ± 0.2
*L. lactis* MG1363	Linares et al., 2010	9.1 ± 0.3
*Lactobacillus plantarum* S-35	Wang et al., 2018	10.3 ± 0.2
*L. plantarum* γ-35	Wang et al., 2018	11.0 ± 0.3
*Staphylococcus aureus* ATCC 29213	ATCC	16.4 ± 0.4
*S. aureus* ATCC 43300	ATCC	16.3 ± 0.4
*S. aureus* ATCC 26112	ATCC	16.1 ± 0.4
*Enterococcus faecalis* ATCC 29212	ATCC	16.2 ± 0.4
*E. faecalis* ATCC 51299	ATCC	8.2 ± 0.2
*E. faecalis* M2	Wang et al., 2018	6.3 ± 0.5
**Gram-negative bacteria**		
*Escherichia coli* DH5α	Takara	0
*E. coli* BL21	Takara	0
*E. coli* BW25113	Grenier et al., 2014	0
*E. coli* JM109	Takara	0
**Fungi**		
*Saccharomyce cerevisiae*	Wang et al., 2018	0
*Pichia pastoris* GS115	Thermo Fisher Scientific	0

### Determination of Physicochemical Characteristics of Subtilin L-Q11

To evaluate the physicochemical characteristics of Subtilin L-Q11, proteolytic, thermal, pH, and surfactant sensitivities of the bacteriocin were tested. To test the proteolytic sensitivity of Subtilin L-Q11, different types of proteases including Pepsin (pH 3.0), Papain (pH 6.5), Proteinase K (pH 7.5), Trypsin (pH 7.6), and Chymotrypsin (pH 7.8), were incubated with the bacteriocin at a final concentration of 1 mg/mL for 3 h. After incubation, the mixture was heated to 100°C for 5 min to terminate the enzymatic reaction. To test its thermal sensitivity, Subtilin L-Q11 was heated to 60, 80, and 100°C, respectively, for 15 and 30 min, and 121°C for 20 min. The pH tolerance of the bacteriocin was measured by adjusting the pH of the buffer to a range of 2–11 using 5 M NaOH or HCl for 2 h. Various chemical reagents, including 1% (v/v) of ethylene diamine tetraacetic acid (EDTA), Tween 20, Tween 80, and urea were incubated with the bacteriocin for 5 h at 37°C to determine its tolerance to the chemical reagents.

After each treatment from above, the antibacterial activity of the residual bacteriocin was measured and compared to the untreated bacteriocin to estimate the loss of the bacteriocin after each of the above treatments.

### Antibacterial Activity of Subtilin L-Q11 Against *S. aureus*

In order to determine the action mode of Subtilin L-Q11, *S. aureus* ATCC 29213 was cultured overnight in TSB broth (BD Biosciences, United States) at 37°C with shaking (200 rpm). Cells were spun down and readjusted to a final density of 10^7^ CFU/mL by using 0.9% sterile NaCl. The final concentration of the bacteriocin was adjusted to 64 μg/mL (MIC_50_ of Subtilin L-Q11 against *S. aureus* ATCC 29213) and the sample with the same amount of TSB broth was used as a control. All the samples for the test were incubated at 37°C under shaking (200 rpm) for 5 h. The cell optical density (O.D._600_) and the number of viable cells were measured every hour (Deraz et al., 2007).

### Scanning and Transmission Electron Microscopy

In order to observe the morphological changes of *S. aureus* ATCC 29213 cells after treated with Subtilin L-Q11, scanning electron microscopy (SEM) was used. *S. aureus* ATCC 29213 cells (1 × 10^7^ CFU/ml) in LB broth were mixed with 256 μg/mL (4 × MIC_50_) of Subtilin L-Q11 and incubated at 37°C for 1, 2, and 3 h, respectively. Cells without bacteriocin treatment were set as a control. Cells in both the bacteriocin treatment and control groups were collected by centrifugation and washed with phosphate buffer (0.1 M, pH 7.0). Cells for SEM observation were fixed for 4 h in 2.5% glutaraldehyde at 4°C and then dehydrated with gradient ethanol solutions (30, 50, 80, 90, and 100%). After that, ethanol was replaced by 100% tertiary butyl ethanol. Cells were then freeze-dried (Wheeler et al., 1975), coated with gold, and imaged using a FEI Quanta 200 SEM.

For observation of intracellular differences between cells in the Subtilin L-Q11 treatment and control groups, transmission electron microscopy was performed as previously described (Yamanaka et al., 2005) with minor modifications. Briefly, cells were fixed as described above. After that, samples for imaging were osmicated in 2% osmium tetroxide at 4°C for 4 h, and then were dehydrated with gradient ethanol solutions as described above. Embedding was performed in epoxy resin at 60°C for 48 h. The sections were then coated with an amorphous carbon film and stained with 2% uranyl acetate and lead citrate. Ultrastructure observation and photomicrographs were then carried out using a JEM-1200EX TEM (Japan Electronics Co., Ltd., Japan).

### Statistical Analysis

Data in this study were presented as means ± standard deviations (SD). One-way analysis of variance (ANOVA) and Duncan’s multiple range test of the data were carried out by SPSS 23.0, and *p* < 0.05 was considered statistically significant. All the experiments were preformed in triplicate.

## Results

### Phylogenetic Characterization of the Strain L-Q11

We performed BLAST search using the DNA sequence of the 16S rRNA gene of L-Q11 as a query against the NCBI GenBank database. Our result indicated that the 16S rRNA gene sequence of L-Q11 showed extremely high similarity (identity ≥ 99%, E value = 0) to *B. subtilis*. We finally classified the strain L-Q11 as *B. subtilis*.

### Dynamic Production of the Antibiotic Against *S. aureus* by *B. subtilis* L-Q11

**FIGURE 1:**
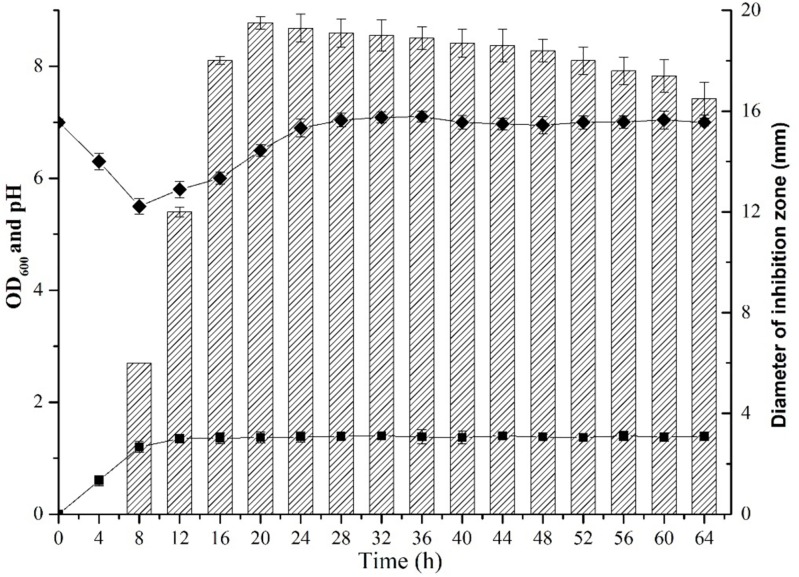
Growth and dynamics of bacteriocin production by *B. subtilis* L-Q11. “
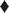
” represents the growth curve of L-Q11; “
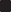
” represents values of pH in the culture medium; “
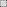
” represents inhibitory activity against the indicator strain *S. aureus* ATCC 29213.

The dynamic profile of antibiotic production by *B. subtilis* L-Q11 during growth was investigated when cells were cultured in LB broth under the shaking condition (200 rpm) at 37°C. The inhibitory activity of the CFS against growth of the indicator strain *S. aureus* ATCC 29213 was used as a measurement of antibiotic production. The growth profile of L-Q11 showed that the culture transitioned to early exponential phase 2 h after inoculation, entered stationary phase at 8 h and remained in stationary phase until end of the experiment (Figure 1). The antibacterial activity in the CFS against *S. aureus* ATCC 29213 was evaluated by measuring the diameter of the inhibition zone. The bacteriocin begun to be produced after cells entered early stationary phase (8 h) and reached maximum at 20 h. Although the antibacterial capacity in the CFS produced by L-Q11 begun to decrease after 20 h, it showed inhibitory activity during the entire stationary phase (Figure 1). The pH of the broth was also measured once every 4 h during the entire experiment. We found that the pH of the medium dropped from 7.0 to 5.2 during the exponential phase and then went back to almost 7.0 (Figure 1). The observed pH changes in the medium indicate that the metabolic capacity of this strain meets the basic requirement of industrial fermentation.

### Purified Bacteriocin From L-Q11 Showed Strong Bacterial Inhibitory Activity

**FIGURE 2:**
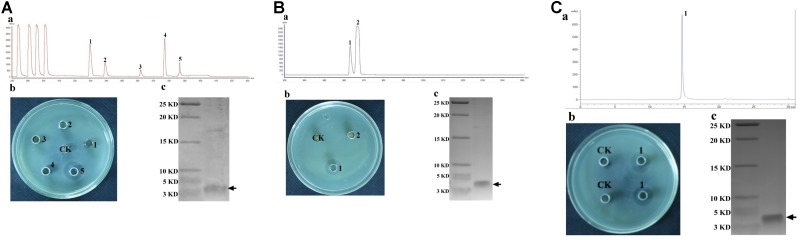
Purification of Subtilin L-Q11 by chromatography. **(A)** cation exchange column; **(B)** gel filtration chromatography; **(C)** RP-HPLC. **(a)** process of purification; **(b)** the assay of antibacterial activity against the indicator strain from the absorbance peaks versus CK (control) by agar well diffusion assay; **(c)** Tricine-SDS-PAGE of the purified active fraction. The arrows indicate the active fraction after each step of purification on Tricine-SDS-PAGE gel.

The CFS collected after 20 h of incubation under the shaking condition was used for bacteriocin extraction and purification by a four-step procedure as described in the Methods. The yield and characteristics of the bacteriocin after each purification step were shown in Table 2. The crude bacteriocin in the CFS was first extracted by ammonium sulfate precipitation and approximately 2-fold enrichment of the bacteriocin was achieved at this stage as measured by the inhibitory activity against *S. aureus* ATCC 29213 (Table 2). For further purification the crude bacteriocin was subjected to a SP-Sepharose Fast Flow cation exchange column. Five main peaks were obtained and separated into five different fractions at this stage. We then tested all five fractions and found that only fraction 5 showed antibacterial activity (Figures 2Aa,b). In addition, we achieved 41-fold enrichment after this step; the antibacterial activity of bacteriocin increased from 1,500 to 37,000 AU/mg against the indicator strain. Next, the active fraction (fraction 5) was injected into a Sephadex G-10 gel-filtration chromatography for the subsequent purification step. During this step, two separate peaks were observed by measuring the absorbance at the wavelength of 220 nm. However, only fraction 1 retained the antibacterial activity (Figures 2Ba,b). Meanwhile, the bacteriocin activity increased 80-fold after this purification step. RP-HPLC was applied in the final purification step of the bacteriocin. The corresponding fraction to this peak showed a strong antibacterial activity against *S. aureus* ATCC 29213 (Figures 2Ca,b). In summary, after series of purification steps, the antibacterial activity of the bacteriocin increased 117-fold compared to that in the initial CFS, reaching 104,000 AU/mg.

**Table 2:** Purification of the bacteriocin produced by L-Q11.

Purification Stage	Volume (mL)	Total protein (mg)	Total activity (AU)	Specific activity (AU/mg)	Purification fold	Recovery (%)
Culture supernatant	200	1156	1,024,000	885	1	100
Ammonium sulfate precipitation	20	531	820,000	1,500	2	80
SP-sepharose fast flow	5	17	612,000	37,000	41	35
Sephadex G10	1	4	313,000	71,000	80	13
RP-HPLC	0.3	1	104,000	104,000	117	2

### Molecular Mass and Amino Acid Sequence Determination

**FIGURE 3:**
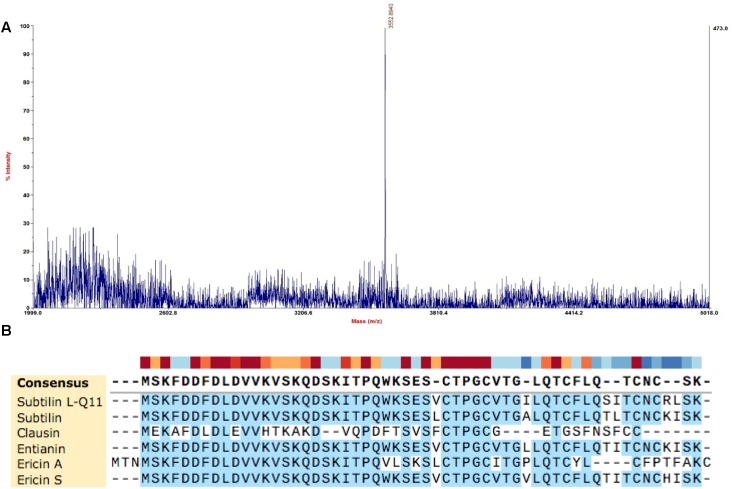
Molecular mass determination and amino acid sequence alignment of Subtilin L-Q11. **(A)** The mass spectrum shown corresponded to the absorbance peak after purification using RP-HPLC in Figure 2C. **(B)** Alignment of published class I bacteriocins from *Bacillus* spp. and Subtilin L-Q11. Alignments were obtained by SnapGene V4.2.6 with default settings.

The molecular mass of the purified bacteriocin produced by *B. subtilis* L-Q11 is 3,552.9 Da as determined by MALDI-TOF MS (Figure 3A). The molecular mass of the bacteriocin produced by *B. subtilis* L-Q11 is different from any of the published bacteriocins. According to the genome sequence analysis of *B. subtilis* L-Q11, the entire amino acid sequence of the bacteriocin was MSKFDDFDLDVV KVSKQDSKITPQWKSESVCTPGCVTGILQTCFLQSITCNCRL SK. Aligned with published bacteriocins from *Bacillus* spp. (Barbosa et al., 2015), bacteriocin produced by *B. subtilis* L-Q11 showed highly similarity to class I bacteriocin but was not identical (Figure 3B). We thus believe that it is a novel Class I bacteriocin and named it Subtilin L-Q11.

### Subtilin L-Q11 Showed a Broad Range of Antibacterial Activities

Subtilin L-Q11 showed a wide range of an antibacterial spectrum (Table 1). It could inhibit the growth of various Gram-positive bacteria including *Bacillus amyloliquefaciens*, *Lactococcus lactis*, *Lactobacillus plantarum*, *Staphylococcus aureus*, and *Enterococcus faecalis.* Some of them are important spoilage bacteria in food industry and human pathogens, such as *S. aureus* ATCC 29213 and species from *Bacillus* genus. However, it showed no inhibitory activity on any of the tested Gram-negative bacteria and fungi (Table 1).

### Subtilin L-Q11 Demonstrated Desirable Thermostability, pH Tolerance, Resistance to Chemical Reagents, and Sensitivity to Proteases

**FIGURE 4:**
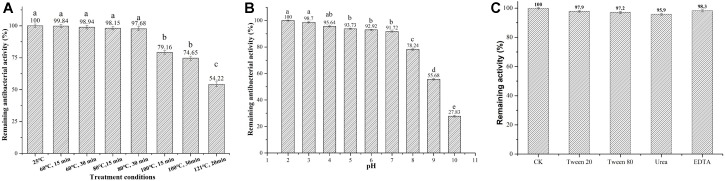
Subtilin L-Q11 demonstrated optimal thermostability, pH tolerance, and resistance to chemical reagents. The effects of pH **(A)**, temperature **(B)** and surfactant **(C)** on the bacteriocin activity produced by L-Q11 were assayed. Relative ratios in percentages were applied to represent retained antibacterial activities of the bacteriocin samples after various treatments when compared to the untreated control group. “abcde” indicates the significant difference among different conditions, the same letter represents no significant differences among those groups and different letters represents significant differences (*p* < 0.05) among those groups.

We next tested several physicochemical characteristics of Subtilin L-Q11, including thermostability, pH tolerance, resistance to chemical reagents, and sensitivity to proteases. In thermostability test, more than 97% of the bacterial inhibitory activity was retained after 15 or 30 min of heat treatment at 60 or 80°C, respectively. Further, the activity was decreased significantly (*p* < 0.05) when under the treatments of 100°C for 15 and 30 min, or at 121°C for 20 min, but there was still more than 54% of activity retained (Figure 4A). Subtilin L-Q11 retained more than 90% of the antimicrobial activity in buffers with the pH ranged from 2 to 7. Although in the buffer of pH 8–10, the antimicrobial activity of Subtilin L-11 decreased significantly (*p* < 0.05), it still showed some activity at pH 10 (27.83%) (Figure 4B). We also tested the impact of several detergents on the activity of the bacteriocin. We found that Subtilin L-Q11 showed great tolerant character after 3 h treatment by 1% (V/V) of Tween-20, Tween-80, Urea and EDTA. It still retained 97.9, 97.2, 95.9 and 98.3% of the activities after treated by above detergents, respectively, (Figure 4C). The activity of the bacteriocin from L-Q11 was totally lost after treatment by any of the three important human digestive proteases, trypsin, chymotrypsin, and pepsin. Also, the commonly used proteinase K could completely eliminate the activity of bacteriocin.

### Subtilin L-Q11 Induced Serious Morphological and Intracellular Changes in *S. aureus* Cells

**FIGURE 5:**
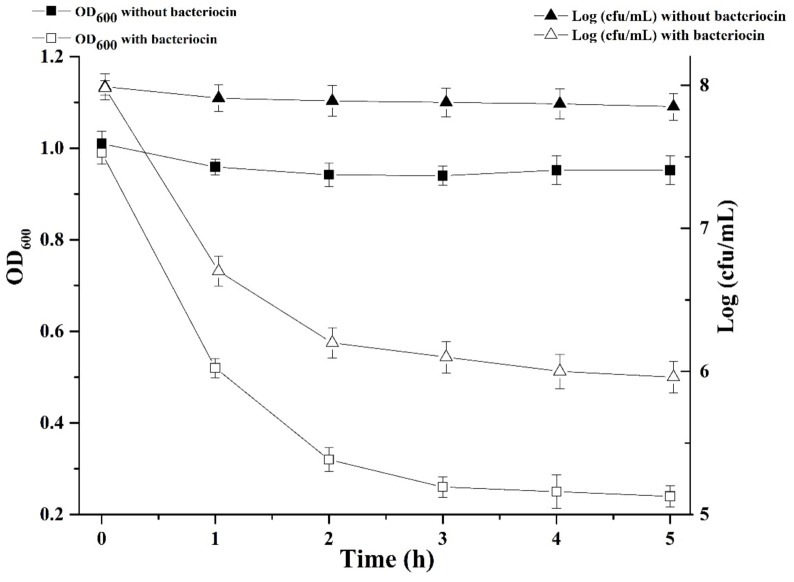
Subtilin L-Q11 treatment triggered cell lysis in *S. aureus*. “
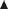
” represents viable cell count of untreated *S. aureus* ATCC 29213; “Δ” represents viable cell count of *S. aureus* ATCC 29213 with the treatment of bacteriocin; “
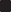
” represents cell optical density at the wavelength of 600 nm (O.D._600_) without bacteriocin treatment; “
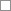
” represents optical density at the wavelength of 600 nm with bacteriocin treatment.

We were interested in better understanding the inhibitory mechanism of Subtilin L-Q11 against *S. aureus* ATCC 29213. Upon treatment of Subtilin L-Q11 for 3 h, the viable number of *S. aureus* ATCC 29213 cells dropped from 8 to 6 [log (cfu/mL)] and the optical density of the culture decreased from 1 to 0.25 (O.D._600_) (Figure 5). This implies that Subtilin L-Q11 induced cell lysis in *S. aureus* ATCC 29213.

**FIGURE 6:**
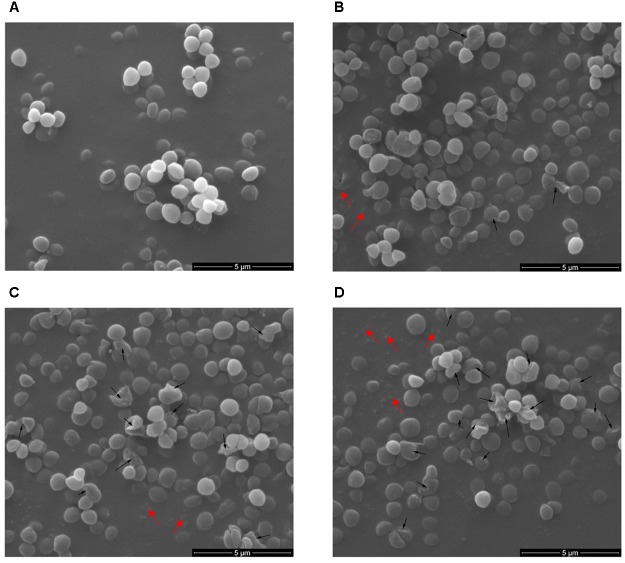
Scanning electron microscopy of *S. aureus* cells treated by Subtilin L-Q11. **(A)** *S. aureus* ATCC 29213 cells from the untreated control group; **(B–D)** *S. aureus* ATCC 29213 cells treated with 256 μg/mL (4 × MIC_50_) of Subtilin L-Q11 for 1, 2, and 3 h, respectively. Scale bars: 500 nm. Black arrows: the cell hollowness and membrane disruption; red arrows: cell fragments. For the sample preparation of TEM and SME, cell cultures were concentrated by centrifuging. During the sample preparation, large amount of the lysed cell (cell fragments) would lost.

To demonstrate in detail, the potential morphological and intracellular changes in *S. aureus* ATCC 29213 cells upon cell lysis caused by Subtilin L-Q11, Scanning Electron Microscope (SEM) and Transmission Electron Microscope were used. Morphological changes in *S. aureus* ATCC 29213 cells were shown in Figure 6. Compared to the regular shape and smooth surface of cells in the control group (Figure 6A), *S. aureus* ATCC 29213 cells treated with 256 μg/mL (4 × MIC_50_) Subtilin L-Q11 for 1 h showed slight hollowness on the surface (black arrows, Figure 6B). After exposed to the bacteriocin for 2 to 3 h (Figure 6C,D), the surface of cells showed more serious hollowness and clear membrane disruption (black arrows). Also fragments of lysed cell were seen in Figure 6B–D (red arrows). Moreover, the bacteriocin-induced deformation of *S. aureus* ATCC 29213 cells happened in a time-related manner.

**FIGURE 7:**
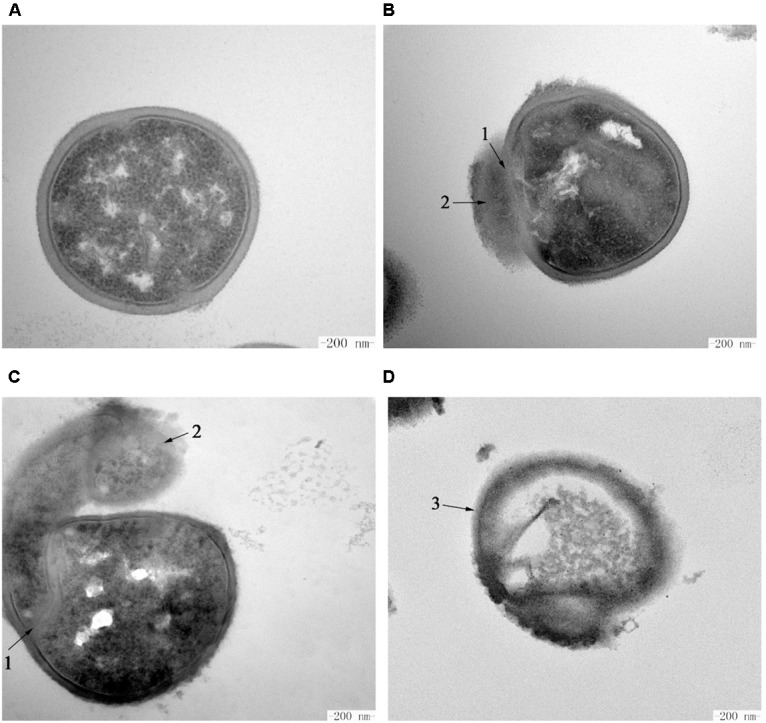
Transmission electron microscopy of *S. aureus* cells treated by Subtilin L-Q11. **(A)** *S. aureus* ATCC 29213 cells from the untreated control group; **(B–D)** *S. aureus* ATCC 29213 cells treated with 256 μg/mL (4 × MIC_50_) of Subtilin L-Q11 for 1, 2, and 3 h, respectively. Scale bars: 200 nm. Arrows numbered 1: the damaged cell membrane. Arrows numbered 2: the leaked intracellular substance. The arrow numbered 3: the ruptured cells.

The intracellular changes of *S. aureus* ATCC 29213 cells treated with Subtilin L-Q11 were observed by TEM. Under TEM, cells in the untreated control group showed features characterized as typical cell wall, and intact and smooth cell membrane (Figure 7A). Also, the biomass and the seemingly genomic DNA were evenly distributed in the cytoplasm. In contrast, after exposure to 256 μg/mL (4 × MIC_50_) Subtilin L-Q11 for 1 h, the intracellular organization of cells was significantly disrupted, showing condensation of genomic DNA and vacuolization (Figure 7B). Moreover, the cytoplasm began to leak slightly (arrow 2 in Figure 7B). When the exposure time was prolonged to 2 h, although the entire cell structure was still largely retained, the seriously disrupted cell membrane was clearly visible as shown in Figure 7C. Also, loss of cytoplasm could be observed in the cells of the treatment group, and the leaked biomass from the cytoplasm could be observed around cell (arrow 1 in Figure 7C). After 3 h of treatment by the bacteriocin, the cytoplasm of the treated cells showed a lower density compared to that of the cells in control group, probably due to the loss of solutes (Figure 7D). Also, a discontinuity and ruptured cell membrane surface of treated cells was shown (arrow 3 in Figure 7D). This indicates that these cells were already lysed. All these observations showed that Subtilin L-Q11 induced serious disruption of the intracellular organization of *S. aureus* ATCC 29213 cells.

## Discussion

In this study, we reported a novel bacteriocin, Subtilin L-Q11, synthesized by *B. subtilis* L-Q11 isolated from orchard soil that demonstrated great biophysical characteristics, which makes it a potential candidate as both an antibacterial compound and a food preservative. A four-step procedure consisting of ammonium sulfate precipitation, SP-sepharose Fast Flow, Sephadex G10 and RP-HPLC, was used for the purification of the bacteriocin. Subtilin L-Q11 retained strong antibacterial activity after purification. According to the genome sequence analysis, the entire amino acid sequence of the Subtilin L-Q11 was determined as MSKFDDFDLDVVKVSKQDSKITPQWKSESVCTPGCVTGILQ TCFLQSITCNCRLSK. The amino acid sequence is similar, but not identical, to the reported Class I bacteriocins (Barbosa et al., 2015). The molecular mass of the purified bacteriocin is 3,552.9 Da, determined by MS. This is different from other known bacteriocins, such as Sentianin (MW = 3446.6 Da) (Fuchs et al., 2011),Ericin S (MW = 3342.8 Da),Ericin A (MW = 2987.7 Da) (Stein et al., 2002), Clausin (MW = 2107.5 Da) (Bressollier et al., 2014), subtilomycin (MW = 3235 Da) (Phelan et al., 2013), Thuricin 4A-4 (MW = 2786.3 Da), and Thuricin 4A-4D (MW = 2886.3 Da) (Xin et al., 2015). We thus believe that the Subtilin L-Q11 produced by L-Q11 is a novel Class I bacteriocin.

For the food industry, pasteurization and high temperature sterilization are the two commonly used strategies for sterilization (Zhao et al., 2016). The thermostability of Subtilin L-Q11 was tested under the conditions of both pasteurization and high temperature sterilization. Our results showed that after treatment it could preserve more than 96 and 58% of the antibacterial activity, respectively, compared to the untreated. Therefore, our newly reported bacteriocin showed great utilization potential in the food industry, especially in the dairy industry. This bacteriocin also showed tolerance to a broad range of pH changes (pH 2.0–9.0), which makes it an excellent food preservative candidate in both acid and alkaline food processing procedures. We also found that the activity of the bacteriocin could be completely destroyed by human digestive enzymes, such as pepsin, trypsin, and chymotrypsin. In other words, the bacteriocin can be decomposed *in vivo* and is thus safe to human health (Hu et al., 2013). Nevertheless, more *in vivo* toxicity experiments should be carried out in the future to confirm the biosafety of the bacteriocin.

We showed that the amount of the important human opportunistic pathogen and food contaminating bacterium *S. aureus* ATCC 29213 decreased 100-fold from 10^8^ to 10^6^ cfu/mL after 3 h treatment by Subtilin L-Q11. The molecular mechanism of the killing of *S. aureus* ATCC 29213 by this new bacteriocin remains unknown. Some of the previously published results showed that one of the major killing mechanisms by some Class I bacteriocins is the disruption of the membrane integrity. Those bacteriocins may cause tiny pores on the bacterial cell membrane (Ennahar et al., 2000; Perez et al., 2014). To further investigate the antibacterial mechanism of Subtilin L-Q11, SEM and TEM were used to show the impacts of Subtilin L-Q11 on the ultra cell structures of *S. aureus* ATCC 29213. The TEM results showed that the intracellular organization of *S. aureus* ATCC 29213 cells upon bacteriocin treatment was seriously damaged, which likely led to cell death (Figure 7). Under SEM, we observed relatively modest changes in *S. aureus* ATCC 29213 cell morphology (Figure 6). Cell membrane damage and leak of biomass from the cytoplasm were clearly observed in bacteriocin treated *S. aureus* ATCC 29213 cells; some cells were even partially lysed. Our observations were also consistent to those previously observed by other bacteriocins. For example, similar cytoplasm damages were reported in nisin and pediocin treated bacteria cells (Kalchayanand et al., 2004; Pattanayaiying et al., 2014). Since membrane damage caused by the pore-forming compounds would lead to severe membrane permeability and leak of the biomass, imbalance of inner and outer membrane (Liu et al., 2017), we believe that the antibacterial mode of action of Subtilin L-Q11 can be further investigated by measuring bacterial membrane potential, intracellular ATP levels, and electric conductivity before and after treatment. Meanwhile, the transcriptomic and proteomic data may also help us to reveal the antibacterial mechanism at the global transcriptional and translational levels. These are ongoing investigations currently in the lab.

## Conclusion

In conclusion, Subtilin L-Q11 not only can inhibit the growth of different types of food-borne pathogens but also show great biophysical characteristics such as thermostability, pH-tolerance, resistance to chemical reagents, and sensitivity to various human proteases. Our results suggest that Subtilin L-Q11 has great potential in both the food industry and the agricultural field, as a new biological food preservative and an antibacterial drug.

## Data Availability

The datasets generated for this study can be found in Genbank, MK156679.

## Author Contributions

YQ, QMS, and PL designed the experiments. YQ, YW, YH, YZ, and QXS performed the experiments. YQ and YW analyzed the results. YQ and YC wrote the manuscript.

## Conflict of Interest Statement

The authors declare that the research was conducted in the absence of any commercial or financial relationships that could be construed as a potential conflict of interest.

## Footnotes

1 http://www.ncbi.nlm.nih.gov/BLAST
